# The Dynamics of the Cell Wall Proteome of Developing Alfalfa Stems

**DOI:** 10.3390/biology8030060

**Published:** 2019-08-19

**Authors:** Kjell Sergeant, Bruno Printz, Gea Guerriero, Jenny Renaut, Stanley Lutts, Jean-Francois Hausman

**Affiliations:** 1 Environmental Research and Innovation (ERIN) Department, Luxembourg Institute of Science and Technology (LIST), 4362 Esch/Alzette, Luxembourg; 2 Groupe de Recherche en Physiologie végétale (GRPV), Université catholique de Louvain, Earth and Life Institute Agronomy (ELI-A), 1348 Louvain-la-Neuve, Belgium

**Keywords:** cell wall dynamism, development, cell wall proteomics, alfalfa, class III peroxidase

## Abstract

In this study, the cell-wall-enriched subproteomes at three different heights of alfalfa stems were compared. Since these three heights correspond to different states in stem development, a view on the dynamics of the cell wall proteome during cell maturation is obtained. This study of cell wall protein-enriched fractions forms the basis for a description of the development process of the cell wall and the linking cell wall localized proteins with the evolution of cell wall composition and structure. The sequential extraction of cell wall proteins with CaCl_2_, EGTA, and LiCl-complemented buffers was combined with a gel-based proteome approach and multivariate analysis. Although the highest similarities were observed between the apical and intermediate stem regions, the proteome patterns are characteristic for each region. Proteins that bind carbohydrates and have proteolytic activity, as well as enzymes involved in glycan remobilization, accumulate in the basal stem region. Beta-amylase and ferritin likewise accumulate more in the basal stem segment. Therefore, remobilization of nutrients appears to be an important process in the oldest stem segment. The intermediate and apical regions are sites of cell wall polymer remodeling, as suggested by the high abundance of proteins involved in the remodeling of the cell wall, such as xyloglucan endoglucosylase, beta-galactosidase, or the BURP-domain containing polygalacturonase non-catalytic subunit. However, the most striking change between the different stem parts is the strong accumulation of a DUF642-conserved domain containing protein in the apical region of the stem, which suggests a particular role of this protein during the early development of stem tissues.

## 1. Introduction

The plant cell wall is a dynamic structure that modulates a wide variety of physiological events and is a key factor in controlling plant phenotype [1]. This dynamism makes it a barrier against stresses [2], whose properties and structure change during development, depending on the environment [3,4]. The plant cell wall is a composite of polymers (cellulose and heterogeneous hemicelluloses), enzymes, and structural proteins embedded in an aqueous gel of pectins with a variable degree of methyl-esterification. Between the plasma membrane and the primary cell wall of some cells, a secondary cell wall is formed, which, in addition to the carbohydrate-based polymers found in the primary cell wall, may contain variable percentages of non-carbohydrate-based compounds, such as lignin, suberin, or cutin. Furthermore, 5–10% of the total mass of the cell wall is made of proteins [5].

The interest in the study of cell wall proteins (CWPs) has been increasing since the 1990s [5]. Since then, and spurred by the increased use of plant biomass in various industrial fields, the understanding of mechanisms underlying cell wall dynamism and organization has increased [6]. CWPs are mainly secreted to the cell wall after recognition of the signal peptide, which destines them to the endoplasmic reticulum (ER). From the ER, CWPs pass through the Golgi complex and the plasma membrane via secretory vesicles. Although the secretory pathway via the ER-Golgi is the most common, alternative secretory routes have been described [7,8]; a process during which proteins undergo specific maturation events, such as N-linked glycosylations and proline hydroxylation [9,10]. However, recent data indicate that, in addition to the classical signal-peptide-based secretion of proteins to the cell wall, the release of extracellular vesicles to the apoplast may form an important contribution to cell wall remodeling [11]. Restructuration of the cell wall during development is performed by the regulated activity of cell wall-located proteins that target xylans [12], polygalacturonans [13], glycoproteins [14], galacto-oligosaccharides [15], cellulose [16], or lignin synthesis [17]. Moreover, proteins with a domain of unknown function (DUF) were identified in cell wall proteome studies (among others DUF26, DUF231, DUF246, DUF248, DUF288, DUF642, DUF1005, DUF1680), all of which may have a function in cell wall development [16,18].

Proteome studies on plant cell walls have been widely performed on the model species *Arabidopsis thaliana.* We recently developed a protocol for the extraction of a cell-wall protein-enriched subproteome by combining protocols optimized on *A. thaliana* and *Medicago sativa* [19,20]. With this protocol, the contamination with intracellular proteins was limited and a high number of cell wall proteins were extracted [21]. Contrary to *A. thaliana*, alfalfa is a crop with an economic interest among others, illustrated by the recent creation of a low-lignin variety [22]. We are currently studying this perennial herb as model for cell wall development in response to different abiotic stresses, for instance long-term cadmium exposure [23,24]. Furthermore, we previously studied the differences in gene expression, protein abundance, and cell wall composition between apical, intermediate, and basal alfalfa stem segments [25]. In particular, total proteome analysis revealed a switch in metabolism between the intermediate and the basal segment. In 2012, Verdonk et al. proposed for the first time a comparative cell wall proteome analysis of developmental stages of alfalfa stems [19]. LC-MS/MS analysis indicated that some proteins varied significantly in abundance. These include peroxidase MtPrx 29, GH family 17 (glucan endo-1,3-beta-glucosidase), or polygalacturonase inhibitor [19]. However, the authors state that the quantitative analysis of this study must be interpreted cautiously since it focused on method development and insufficient replicates have been analyzed. Other studies on the cell wall proteome of stems include, among others, studies on *A. thaliana* [26], sugarcane [27], and flax [28]. Given the current interest in the cell wall proteome, several reviews on the topic have been published, for instance focusing on crops [29]. The abundance of post-translational modifications, such as proline hydroxylation, didehydrophenylalanine, and protein O-glycosylation [10,30,31] and their impact on the function of cell wall localized proteins is a topic of intense research among others for the use of plant systems for protein production.

Here, we describe the experimental work and data from a study aimed at unravelling the significant changes occurring in the cell wall proteome of differently aged tissues, corresponding to different heights along the alfalfa stem. Since the stem of herbaceous plants shows a basipetal lignification gradient accompanying age-dependent progressive secondary growth, the different regions sampled along the stem axis correspond to distinct stages of cell wall maturation. This sampling strategy sheds light on the cell wall dynamics of the growing stem. In summary, our analysis indicates that in the apical region xyloglucan endotransglucosylases, stem 28 kDa glycoprotein, germin-like proteins, DUF642-proteins, and class III peroxidase MtPrx15 are highly abundant. Beta-galactosidases are more abundant in the apical and the intermediate regions. In contrast, endochitinases, aspartic proteinases, peptide-asparagine amidase A (PNGase), class III peroxidases MtPrx29, MtPrx41, and lectins are highly abundant in the mature, basal region. Those proteins that have a significant change in abundance are discussed according to their implication in cell wall metabolism.

## 2. Materials and Methods

### 2.1. Plant Material

Soil was collected in a local field (49°33’44′ N, 5°41’49′ E, Musson, Belgium), dried, mixed, sieved at 5 mm, and used to fill 48 containers (7.5 cm × 7.5 cm × 10 cm). Alfalfa (*Medicago sativa* L. cv Giulia) seeds were inoculated with a peat-based inoculant (HiStick^®^, Becker Underwood), according to the manufacturer’s instructions, and sown 3 seeds per pot. Plants were grown in incubators (Fitotron SGC 120, Weiss Technik UK, Leicestershire, UK) at 22 °C/17 °C, 13 h/11 h d/n, 60% humidity. Following germination, 2 of the 3 plants per container were selected and watered with deionized water. A first cut was performed 2 months after sowing, and plants were allowed to regrow. Soils were then weekly fertilized with 50 mL macronutrient solution (Hoagland 1X macronutrients only) [32]. All growing shoots reaching 25 cm and developing at least 9 internodes were sampled 6 weeks after the first cut. Stems were separated from leaves and stipules with a razor blade and divided into 3 parts of equal length, namely the apical, intermediate, and basal regions. In a previous study [25] the stem was divided into five parts and part 1/5, 3/5, and 5/5 were analyzed to have more discriminative samples. The requirement of larger samples for cell wall protein enrichment necessitated the application of a different sampling strategy. For each stem segment, 4 individual biological replicates (4 g to 5 g for fresh weight) were prepared and stored at −80 °C prior to protein extraction.

### 2.2. Cell Wall Protein Enrichment

#### 2.2.1. Extraction of Cell Wall Proteins

Cell wall enrichment was attained using a sucrose gradient procedure as described by Feiz et al. [20]. Protein extraction was done with a sequential extraction method using buffers supplemented with CaCl_2_, ethylene glycol-bis(β-aminoethyl ether)-*N,N,N′,N*′-tetraacetic acid (EGTA), and LiCl [21].

The frozen plant material was ground in liquid nitrogen and transferred to a 50 mL Falcon^®^ tube with 20 mL 0.4 M sucrose, 5 mM Na^+^ acetate, pH 4.6 at 4 °C. After vigorous shaking (2 min at 25 Hz), the samples were placed on a rocking platform (overnight, 4 °C) and subsequently centrifuged (1000 *g*, 15 min, 4 °C). After centrifugation, the supernatants were discarded and the pellets were resuspended in 10 mL of the same buffer, but with 0.6 M instead of 0.4 M sucrose. After 30 min on a rocking platform at 4 °C, the samples were centrifuged (1000 *g*, 15 min, 4 °C) and the supernatants were again discarded. This washing step was repeated respectively with the same buffer with 1 M sucrose and, subsequently, twice with the buffer without sucrose. The pellets, corresponding to the cell wall-enriched samples, were transferred to 30 mL tubes.

To these cell wall-enriched samples, 7.5 mL 5 mM Na^+^ acetate, 200 mM CaCl_2_, and pH 4.6, 4 °C was added, and the samples shook on a rocking platform for 30 min at 4 °C. After centrifugation (10,000 *g*, 15 min, 4 °C), the supernatants were saved, the procedure repeated, and supernatants pooled. These pooled supernatants formed the CaCl_2_ fraction.

A total of 10 mL 5 mM Na^+^ acetate and 50 mM EGTA (pH 4.6) was added to the pellet, which shook vigorously for 1 h at 37 °C. After centrifugation (10,000 *g*, 15 min, 4 °C), the supernatants were saved and the incubation with EGTA was repeated twice. The EGTA fraction is composed of the pooled supernatants of these three repetitions.

A total of 15 mL 5 mM Na^+^ acetate and 3 M LiCl, (pH 4.6) was added to the pellet after EGTA extraction, The pellet was resuspended and placed overnight on a rocking platform (4 °C). The supernatants after centrifugation (10,000 *g*, 15 min, 4 °C) were saved, forming the LiCl fraction.

#### 2.2.2. Concentration and Desalting of the Extracts.

Amicon Ultra-15 10K Centrifugal Filter Devices (Millipore) were used to reduce the sample volume to approximately 200 µL (4700 *g*, 4 °C). This final volume was washed and desalted using the Bio-Rad ReadyPrep 2-D Cleanup Kit. Finally, the dried protein samples were solubilized in 100 µL labelling buffer (7 M urea, 2 M thiourea, 2% (w/v) CHAPS, 30 mM Tris), and the Bradford protein assay with bovine serum albumin as standard was used to determine the protein concentrations.

### 2.3. Cell Wall Protein Analysis by 2D-DIGE

#### 2.3.1. Protein Migration

Distinct gel images were obtained for the three factions. Therefore, these were analyzed separately, using a separate internal standard and separate analysis. Labelling, migration, and spot detection were performed as previously described [25] with the following slight modifications: (1) 50 μg of proteins for the CaCl_2_-fractions, 40 μg of proteins for the EGTA, and 40 μg of proteins for the LiCl fractions were labelled with respectively 400 pmol and 320 pmol of the cyanine NHS-dyes LUMIPROBE LLC (Hannover, Germany); (2) following labelling, resolubilization in the lysis buffer and an addition of Destreak Reagent (GE-Healthcare, Chiago, IL, USA) and Ampholyte (Bio-Rad, Hercules, CA, United States) (pH 3–10) samples were loaded onto Immobiline^TM^ DryStrip (3–10 NL, 24 cm) for overnight rehydration; (3) a 5 step program was used for isoelectric focusing (IEF): (1) 100 V for 3h, (2) a 4 h linear gradient from 100 V to 1000 V, (3) 1000 V for 6 h, (4) linear gradient from 1000 V to 10,000 V for 6 h, and (5) 10,000 V till 95,000 Vh were reached. During IEF, the current was limited to 75 μA per strip. The use of different protein quantities for the different fractions has no influence on normalization and quantifications since the three fractions were analyzed independently.

#### 2.3.2. Spot Selection

Following spot detection and matching using Decyder (v7.0, GE-Healthcare), the normal volume of each spot was extracted, and statistical analysis was performed using R (v3.1.2) with the package FactoMineR v1.0. Prior to statistical analysis, the normal spot volumes were standardized by dividing the volume of each spot by the corresponding spot volume of the internal standard. Spot average ratio *r* (Apex vs Intermediate, Apex vs Base, and Intermediate vs Base) was calculated. In case this *r* ratio was inferior to 1, the additive inverse of the inverted r (−1/r) is reported. Data (standardized spot volumes) were further log2 transformed. Only spots with no more than 1 missing value per condition were kept for analysis. Comparisons (Apex vs Intermediate, Apex vs Base, and Intermediate vs Base) were performed by spot, using a two-tailed Student t-test for samples of equal variance. Only spots with a r-value <−1.5 or >1.5 and *p*-values inferior to 0.05 were considered to vary significantly. Spots of interest were visually checked using the Decyder (v7.0, GE-Healthcare) spot view and selected for picking.

#### 2.3.3. Spot Picking, Digestion, MS/MS Analysis

Selected spots were picked with an Ettan Spot Picker (GE- Healthcare). Digestion was carried out using a Freedom EVO II workstation (Tecan, Männedorf, Switzerland). Briefly, gel plugs were washed with 50 mM ammonium bicarbonate in 50% (v/v) methanol/MQ water and dehydrated with 75% acetonitrile (ACN). After dehydration, proteins were digested with trypsin Gold (Promega, Fitchburg, WI, USA) and 8 µL of a solution containing 5 ng/µL trypsin in 20 mM ammonium bicarbonate (overnight, 37 °C). After digestion, peptides were extracted from the gel plugs with 50% (v/v) ACN/0.1% (v/v) trifluoroacetic acid and dried. Peptides were solubilized in 0.7 µL of 50% (v/v) ACN/ 0.1% (v/v) TFA and spotted on MALDI target plates. To this, 0.7 μL matrix (7 mg/mL α-cyano-4-hydroxycinnamic acid in 50% (v/v) ACN/0.1% (v/v) TFA) was added.

A peptide mass spectrum was acquired using the 5800 MALDI-TOF/TOF MS (Sciex, Darmstadt, Germany), the 10 most abundant peaks, excluding known contaminants, automatically fragmented. MS analyses were carried out as described by Printz et al. [33]. MS and MS/MS spectra were submitted for database-dependent identification against the NCBInr database limited to the taxonomy *Viridiplantae* (1,717,798 sequences) (http://www.ncbi.nlm.nih.gov) on an in-house MASCOT server. A second search was done against an EST Fabaceae database (19,932,450 sequences). The parameters used for these searches were mass tolerance MS 100 ppm, mass tolerance MS/MS 0.5 Da, maximum 2 missed cleavages, fixed modification carbamidomethyl-cysteine, variable modifications oxidation of methionine, oxidation of tryptophan kynurenine, and double oxidation of tryptophan. Proteins were considered identified when at least two peptides passed the MASCOT-calculated 0.05 threshold scores (Supplementary Table S1A–G). All identifications were manually validated. When high quality MS/MS spectra were not identified in database searches, the sequence of the peptides was determined manually. In order to obtain an objective confidence score for these peptides, the spectra were resubmitted with adjusted search parameters. After manual validation, these were added in the Supplementary Table S1A–C with the peptide score in red. When peptides in the same spot matched different database entries, they were aligned, and it was verified that these belong to the same protein using the NCBI BLAST and the Alfalfa Gene Index and Expression Atlas Database (AGED) databases (http://plantgrn.noble.org/AGED/). When a protein with a trivial name was identified, a BLAST alignment against the *viridiplantae* database was done and the name of the protein with the highest significant homology was added in the result table. For the prediction of the localization of the proteins, the recently developed algorithm DeepLoc was used [34].

#### 2.3.4. Post-Identification Statistical Procedure

Spots containing 1 significant protein (116) were analyzed using the freeware R (v3.1.2) with the additional package FactoMineR v1.0. Principal component analysis (PCA) was performed on this set of proteins (Supplementary Table S2A). PCA was carried out with the R-function PCA by using the normalized spot volumes of the 116 spots in each stem region. Proteins were clustered in 10 groups with the hierarchical clustering on principle components (HCPC). The R function and PCA axis were characterized using the *dimdesc* function of the FactoMineR v1.0 package [35]. This procedure allowed the extraction of a set of 103 proteins that highly contribute (*p* < 0.05) to Dim1 and/or Dim2, among which 61 (61.6%) and 4 (66.7%) proteins were predicted to be secreted (Supplementary Table S2B,C). Peroxidases were identified using the web-tool PeroxiBase, available at http://peroxibase.toulouse.inra.fr/ [36]. Multiple sequence alignment was performed using Clustal Omega, available at: http://www.ebi.ac.uk/Tools/msa/clustalo/.

## 3. Results

The cell wall-enriched proteome of the stem of alfalfa (1st year of growth, 2nd cut from plants with 3 to 4 shoots) was extracted from three different stem regions: The apical, intermediate, and basal stem. CWPs were extracted according to a protocol optimized on alfalfa stems and derived from previous work on plant cell wall proteomics [19,20,21]. This procedure includes a first step of cell wall enrichment followed by a 3-step sequential extraction procedure with buffers containing CaCl_2_, EGTA, and LiCl. Each of these fractions was analyzed separately, and normalization and the following quantification was done for the CaCl_2_, EGTA, and LiCl fractions independently. Four biological replicates were generated for extraction. Proteins were labelled using fluorescent dyes and separated on 2D-gels (2D-DIGE) prior to quantitative analysis and protein identification (Supplementary Figure S1), as previously done by Watson et al [37]. Since no current proteomics techniques allow the analysis of all proteins present in a sample, the here-reported results correspond to that part of the proteome that can be analyzed with the used approach and is limited by the extraction protocol and the use of 2D-DIGE for visualization and quantification. The intracellular proteins identified are reproducibly found and their abundances changed significantly. They are part of the subproteome that is extracted and thus are discussed as such. The big groups of proteins identified in this study are similar to those found in other cell wall proteome studies [27,38,39].

Only spots matched on 75% of the gels and displaying a volume ratio *r* superior to 1.5 or inferior to −1.5 (Student t-test *p*-value <0.05) were considered as significant. According to these criteria, 91 significant spots were selected and analyzed in the CaCl_2_ fraction, 139 in the EGTA fraction, and 27 in the LiCl fraction. The MS analysis of these 257 spots resulted in 186 significant identifications, among which 122 (65.6%) were predicted to carry a signal peptide (SignalP) [40]. Since the presence of more than one protein in a spot excludes the quantification of the individual protein, only the 116 spots, wherein exactly one protein was identified, can be used for biological interpretation. Given that a different location on the gel (change in pI and/or molecular weight) of two spots, nominally containing the same protein, indicates that these are either isoforms encoded by different, homologous genes or different forms of the same gene product due to post-translational processes or artefactual changes, which are treated separately. The use of DeepLoc on these 116 spots resulted in the prediction of secreted, soluble protein for 65 (56%) spots. Although Deeploc performs well in predicting the subcellular localization of proteins, only the actual observation of a protein outside of the plasma membrane can confirm this. The change of one amino acid or, for instance, saturation of the vacuolar sorting system [41] are among the factors that may influence the actual localization of a protein.

These cell wall-predicted proteins were classified based on previously listed functional classes [42]. This classification was performed according to the list of domain hits proposed by the NCBI blast web-tool and from available literature (Supplementary Table S2D). The most represented class of proteins that undergo significant variations in abundance along the stem of alfalfa is “proteins acting on carbohydrates”. The other proteins are essentially classified as “oxido-reductases”, “proteases”, “proteins with interaction domains”, “proteins with nutrient reserve function”, and “miscellaneous proteins”. The highest fold-change of the study (7.56, Apex vs Base) was found for an unknown protein (NCBInr gi:537313) 98% identical in sequence with the *Medicago truncatala* plant F18G18-200 protein (CM001217.2) with a DUF642 conserved domain that was identified in four spots (two in the CaCl_2_ fraction (1586 and 1609) and two in the EGTA fraction (956 and 965)). In the following discussion and tables, the name F18G18-200 is used for this protein. The protein identified in spot CaCl-2827 is 90% identical in sequence to acidic endochitinase and discussed as such.

**Figure 1:**
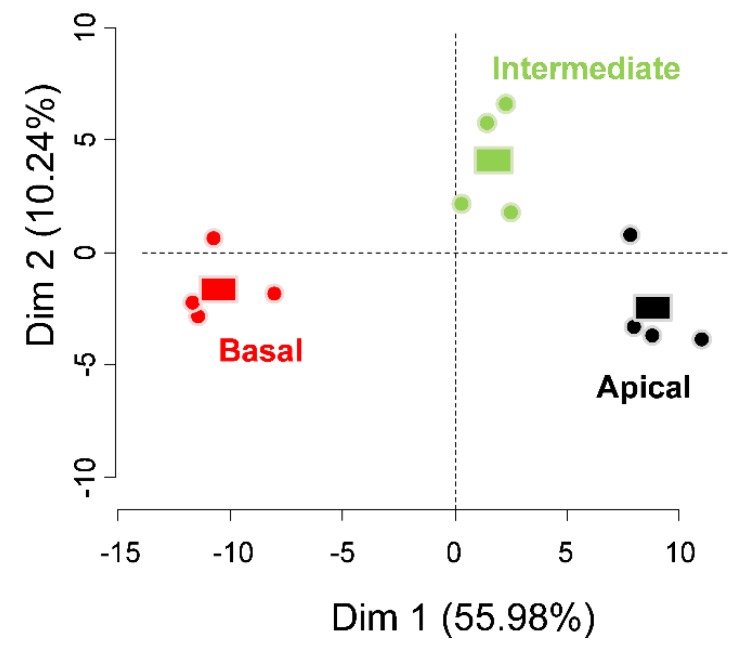
Principal component analysis (PCA) showing the distribution of the individuals based on the set of 113 proteins differentially regulated in the different stem regions. Only the two first components are represented (PC1: 55.98%, PC2: 10.24%). Each dot refers to one replicate and squares represent the barycenter of the dots corresponding to one stem region.

PCA followed by post-PCA clustering in 10 groups was performed on the set of 116 spots, in which only one protein was identified (Figure 1 and Table 1). The first component (Dim 1) of the PCA explains more than half (55.98%) of the variability of the cloud of points and allows us to differentiate the three stem regions, indicating that each stem segment has a characteristic cell wall proteome. Similar discrimination was obtained in a non-targeted proteome study of the same three stem regions [25]. The highest similarities were observed between the apical and intermediate stem regions. The second dimension of the PCA accounts for 10.24% of the variability and differentiates the intermediate from the basal and apical stem regions. However, this dimension is represented by only six spots, which significantly contribute to this axis (*p* < 0.05), indicating that few proteins had a behavior specific to this segment. From the 116 proteins that changed significantly, a subset of 103 proteins was identified as “high contributors” to these two PCA-axes. These were extracted using the *dimdesc* function of ‘FactoMineR’ at the threshold of 0.05 (Supplementary Table S1B,C). From this selection, two distinct trends emerged. The proteins either accumulated more in the apical (49 proteins) or basal (50 proteins) part of the stem. Only four proteins, all from the cysteine protease family (CaCl_2_ 2269, EGTA 1450, EGTA 1452, and EGTA 1451) accumulated mainly in the intermediate stem region.

Proteins showing a higher accumulation in the most lignified basal region were grouped mainly in clusters 1 to 4 (Table 1) and were identified, among others, as members of the lectin family: PNGase, chitinases/endochitinases, and glucan endo-1,3-β-glucosidases (Table 1 and Supplementary Table S2D). Proteins not predicted to be cell wall localized also grouped in these clusters, especially ferritins, beta-amylases, and glutelins type-A (Table 1 and Supplementary Table S2D). In contrast, proteins belonging to groups 6 to 10 were more abundant in the young, apical segment. The most remarkable changes in comparison with the base of the stem were the high abundance of beta-galactosidases, germin-like proteins, class III peroxidases, plant/F18G18-200 proteins, stem 28 kDa glycoproteins, polygalacturonase non-catalytic proteins, and xyloglucan endotransglucosylases. Confirming findings that chloroplast-located proteins are more abundant in the green, apical region, it was found that non-cell wall proteins from these clusters are chloroplast localized. We further noted the higher abundance of one dirigent-like protein (Pfam 03018), putatively involved in stereoselective lignan biosynthesis (Table 1).

**Figure 2:**
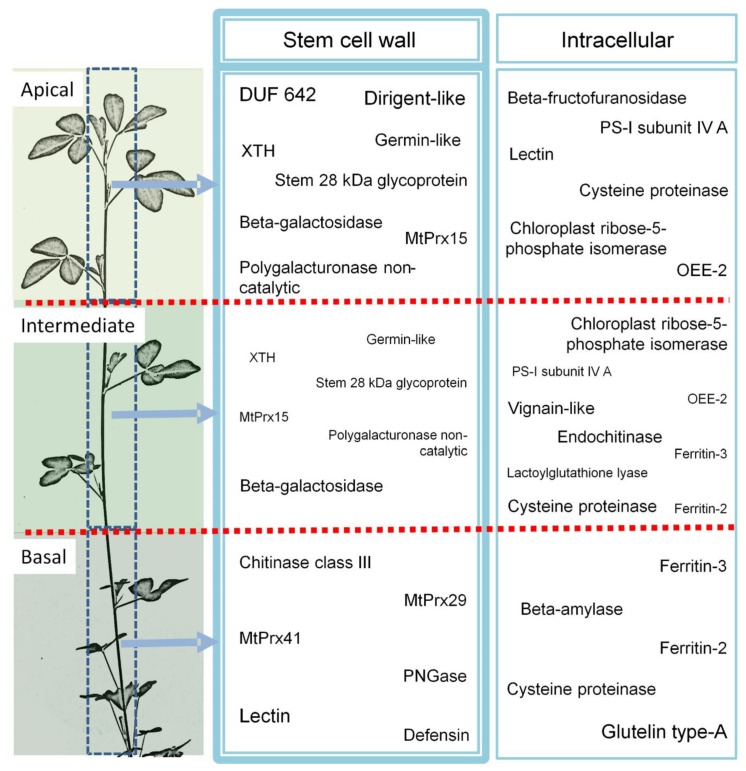
Profiling of the CWP and non-CWP (intracellular) discussed in this study and identified in the apical, intermediate, and basal stem region (big font size = high abundance, medium font size = medium abundance).

The abundance of these 103 high contributor proteins in the intermediate stem region was generally intermediate between that in the two extreme stem parts. However, proteins from cluster 7, which contained 18 isoforms of beta-galactosidase, accumulated to a similar extent in the apical and intermediate region. Proteins from cluster 1, containing mainly lectin-like proteins, accumulated in the basal segment and had a lower abundance in the younger stem parts. In contrast, the two DUF642 proteins (Plant/F18G18 in Table 1) from cluster 10 strongly accumulated in the apical part. Figure 2 summarizes the most important changes occurring along the alfalfa stem identified in this study.

During MS analysis, several post-translational modifications were observed and are added in the Supplementary Table S1A–C, most of which concern the confirmation of predicted signal peptides. The proteolytic cleavage of N-terminal presequences or inhibitor domains (for instance the spots 914, 969, and 1233 in the EGTA fraction) was confirmed by a literature search or blasting the found sequence and identification of the active domain. Finally, α-β dehydration of phenylalanines in the beta-subunit of polygalacturonase (LiCl spot 300), as recently described [30], was confirmed.

A C-terminally truncated calreticulin was observed (CaCl_2_ spot 1274). Although regarded as an ER-resident protein, calreticulin was described in other subcellular compartments [43]. Animal calreticulin is linked with the recognition of tumor and apoptotic cells, and wound healing in plants it is linked to growth and stress responses [44]. Although a mass loss of approximately 6 kDa was observed in vitro [45], to our knowledge, cleavage in the C-domain of calreticulin was not yet observed in vivo. The found cleavage site DPVD is conserved in plant homologues and corresponds to a caspase-like activity, a regulatory proteolytic activity that is known in plants [46]. Although our data explains the lack of immunological detection of the C-terminal HDEL-sequence for plasma membrane-linked calreticulin [47], a further study should clarify whether this observation stems from processing or degradation. 

## 4. Discussion

Uniform stems of alfalfa were dissected in equal thirds and cell wall protein-enriched fractions extracted using a sequential extraction protocol. The different sub-proteomes were independently analyzed using a gel-based approach. This quantification strategy was selected because the fractions contain relatively few different proteins, thus limiting the masking effect when 2D gels are used on more complex samples. Furthermore, a separation of proteins, and not peptides as in LC-MS-based approaches, allows visualizing the heterogeneity of the different forms, in which a gene product can be present in a sample. This has the drawback of having more than one spot and thus a quantitative value for proteins that are heterogeneous, hindering straightforward interpretation. However, this heterogeneity is biological. A way to overcome the inherent limits of gel-based proteomics while keeping the capacity to visualize all different forms in which a protein is present is the use of top-down proteomics, an approach not yet ready for application on the system studied here [48].

### 4.1. Carbohydrate-Interacting Proteins Accumulate in the Cell Wall of the Apical Region

The cell wall of the apical region is characterized by a significantly higher presence of a stem 28 kDa glycoprotein (EGTA-1391), a polygalacturonase non-catalytic protein (LiCl-300), and xyloglucan endoglucosyltransferase (Glycoside Hydrolase 16 (GH16)—CaCl_2_-1945 and CaCl_2_-1940), which cluster in groups 8 and 9 (Table 1). In the alfalfa stem, we previously observed that the abundance of this 28 kDa glycoprotein varied significantly with changes in copper availability in the substrate [49], and Verdonk et al. also observed a higher accumulation of this protein in the apical region [19]. In particular, it accumulated more in the apical region when copper availability was optimal. Here, the protein is found in the highest abundance in the youngest stem region (containing elongating internodes), which correlates with a high expression level of the contig 59881 (plantgrn.noble.org/AGED). This corroborates the attribution of a specific role to this protein during the early growth of the stem [50].

The higher abundance of the polygalacturonase (PG) non-catalytic protein (β-subunit) (LiCl-300) is consistent with the higher expression of contigs coding for this protein. The data on the contigs 111493, 12933, 27771, 48302, 51002, 22692, 53836, 12932, 12931, and 65583—all coding for isoforms of this protein—as can be found at http://plantgrn.noble.org/AGED/, indicate a higher expression in younger tissues. The expression data thus reinforces our data, indicating that these proteins are involved in early stem development. The function of these proteins remains elusive. Tomato plants expressing a PG β-subunit antisense transgene show an increased degradation of the pectin fraction of the cell wall [51]. It was thus hypothesized that the presence of the β-subunit restricts pectin solubilization and limits pectin depolymerization during fruit ripening. In particular, the β-subunit may protect or limit the access of the catalytic PG protein to pectin sites within the cell wall. The hypothesis that PG β-subunit modulates in vivo PG activity was supported by findings on wound-inducible PG β-subunit antisense tomato plants, which resulted in elevated PG activity [52]. In the young tissues, such as the elongating stem of alfalfa, the high abundance of PG non-catalytic proteins may thus limit pectin degradation.

The two xyloglucan endotransglycosylase/hydrolase (XTH) (CaCl_2_-1945 and CaCl_2_-1940) share respectively 80% and 79% identity with *A. thaliana* protein AT2G06850 and 86% with VrXTH1 from *Vigna radiata*. In the *A. thaliana uro* mutant, with delayed secondary cell wall formation, the expression of XTH-coding genes (including AT2G06850) is significantly reduced [53]. In *V. radiata,* the VrXTH1 gene is inducible by brassinosteroids, auxin, and Ca^2+^ and is highly expressed in the hypocotyl and stem, more specifically in the epidermis and cortical layers, where cell elongation occurs [12,54]. XTH4 catalyzes the hydrolysis of ß(1–4) bonds in the xyloglucan backbone and transfers the xyloglucanyl segment to the non-reducing glucose residue of an acceptor [55], being a xyloglucan or a xyloglucan oligosaccharide. This protein may thus ease cell growth by modifying the cell wall structure through cleavage and rejoining of cross-links between xyloglucan and microfibrils. The high abundance of these proteins in the apical stem region is in agreement with observations done in *V. radiata* hypocotyls [12].

Young stem tissues are also characterized by the high abundance of DUF642-domain proteins, with an unknown function (NCBInr gi:537313, homologous to Medtr1g011800.1) [56]. In 2016, it was mentioned that proteins with this domain were found in all cell wall proteome studies [27]. These proteins were identified in both the CaCl_2_ (spots 1586 and 1609) and EGTA (spots 956 and 965) fraction and underwent the most important change in accumulation in this study, indicating a role of DUF642-domain proteins in the development of younger stem segments. In *A. thaliana*, BIIDXI, a DUF642 protein, is linked with hypocotyl growth via the efflux of auxin [57]. Whether other DUF642 proteins have similar functions remains to be determined. In a study on the development of etiolated *A. thaliana* hypocotyls, few changes were observed in the accumulation of five proteins with a DUF642-domain [38]. For at least some isoforms of DUF642-proteins, in vitro interaction with cellulose and, to a lower extent, hemicellulose, has been observed [16]. Phylogenetic analyses have shown that this domain is specific to the angiosperm and gymnosperm lineage [16]. The differential expression of DUF642 genes in different plant organs and at different time points indicates the existence of a tight spatiotemporal regulation of these genes and suggests that different members may exert a distinct role in plant development [58,59].

**Figure 3:**
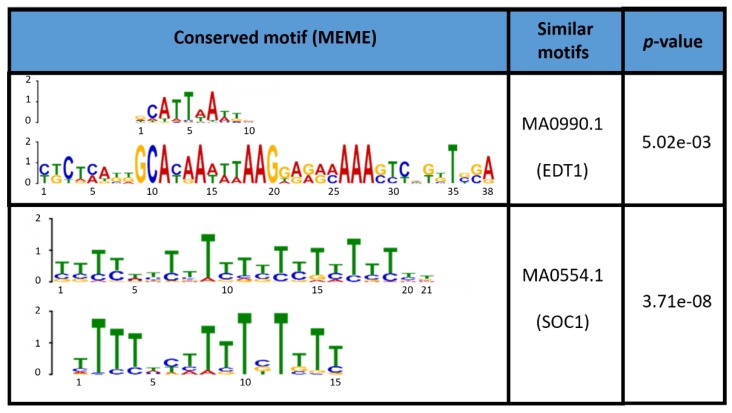
Conserved motifs in the promoters of the seven DUF642 genes from *M. truncatula*. The 1000 bp upstream of the start codon were analyzed using the MEME suite 4.11.2. The first motif in each table cell is the one found in the database; the second motif is the one found in the promoters of alfalfa genes. The *p*-values are indicated.

Doing an in silico analysis, we identified 7 *M. truncatula* genes coding for homologous DUF642 proteins (Medtr1g011800.1, Medtr2g103170.1, Medtr0045s0110.1, Medtr4g039680.1, Medtr4g039720.1, Medtr4g039740.1, Medtr2g019600.1). All seven genes have one to six GTAC-motifs in their 1000 bps 5’-UTR promoter region, a motif which may be recognized by the SQUAMOSA promoter-binding protein-like 7 (SPL7) transcription factor, the central regulator of Cu-homeostasis in plants [60]. In addition to that, a promotor analysis of these seven genes revealed two conserved motifs (Figure 3). Using the JASPAR CORE 2016 plants database resulted in the identification of EDT1/HDG11 and SUPPRESSOR OF OVEREXPRESSION OF CONSTANS 1 (SOC1) as candidates for binding to these promoter-motifs. EDT1/HDG11 is a HD-ZIP IV transcription factor upregulating cell wall-loosening genes [61]. Therefore, DUF642 may be regulated via the same HDG11-dependent transcriptional circuit and may partake in elongation in young stem regions. (SOC1) is a MADS-box transcription factor involved in flowering time control. When induced in the shoot apex, it regulates (via gibberellin interaction and photoperiod perception) the transition from vegetative to reproductive growth. In this respect, it should be noted that SPL3 is directly regulated by SOC1 [62]. Furthermore, some members of the DUF642 protein family directly interact with pectin methylesterase (PME) isoforms, generating the hypothesis that they modulate the pectin methylesterified state in vivo and are thus involved in the fine-tuning of the biomechanical properties of the cell wall [18,58]. The involvement of DUF642 proteins in cell elongation in young tissues was recently shown [63], and the identification of these conserved promoter motifs as binding sites for transcription factors involved in cell wall loosening may contribute to the further disentanglement of their physiological function.

### 4.2. β-galactosidases are Abundant in the Apical and Intermediate Stem Part

Two β-galactosidase proteins (NCBInr gi:657400954 (one spot) and NCBInr gi:357437609 (18 spots), Glycoside Hydrolase 35), sharing respectively 70% and 67% identity with the *A. thaliana* homologue AT4G26140.1 (Gal-12, β-s subfamily a1), accumulate more in the intermediate and apical region compared to the basal segment. A similar increased accumulation of beta-galactosidase was previously observed in the younger tissues of alfalfa stems compared to older internodes [19]. Moreover, the higher expression of genes coding for β-galactosidases in young tissues was previously observed. For instance, Iglesias et al. (2006) report a higher expression of AT4G26140 in young leaves and apical stems compared to mature leaves and the basal stem region [64]. Likewise, a transcriptome study on alfalfa found a more than five-fold higher expression of beta-galactosidase genes in younger compared to older tissues [65]. As all members of this family, Gal-12 hydrolyses β-(1,4)- and β-(1,3)-linked galacto-oligosaccharides, such as those found in the pectins galactobiose and galactotriose. However, since this protein is unable to cleave galactose from xyloglucan oligosaccharides, larch wood arabinogalactan, or oat xylan, it is unlikely to be implicated in hemicellulose remodeling [15]. In our study, high accumulation of β-galactosidases is observed in the apical and intermediate stem region, confirming its implication in the youngest regions. Since mature root sections labelled with Gal-12 antibodies showed specific labelling in thickened walls of xylem cells, its homologue, which accumulates in young alfalfa stem regions, may act in the remodeling of the xylem cell wall.

### 4.3. Class III Peroxidases MtPrx29, MtPrx41, and MtPrx15

Whereas most CWPs from the same family follow similar variations in abundance, different members of the class III peroxidase family (Class III Pfam PF00141) behave differentially. Similar disperse changes in abundance during cell wall maturation were previously observed [38]. Proteins identified with the two NCBInr identifiers gi:357491415 (MtPrx29/MsPrx05—cluster 5, CaCl_2_-1769) and gi:657377089 (MtPrx41—cluster 4, EGTA-1168) accumulate more in the basal region, an observation previously made when developing alfalfa [19]. The corresponding contigs (contig 65262, 88968, and 16417) have a higher expression in post-elongating internodes. In contrast, the three spots wherein the protein was identified with the NCBInr identifier gi:537317 (MtPrx15/ MsPrx15—cluster 8, LiCl-340, LiCl-1156, LiCl-1163) are of higher abundance in the apical region, and since they are identified in the LiCl extract, they interact more strongly with the cell wall. Class III peroxidases belong to a multigenic protein family, involved in processes such as limiting the spread of infections to the strengthening of the cell wall [66]. During plant growth, cell expansion relies on a balance between cell wall stiffening and cell wall loosening, antagonistic processes wherein members of the class III peroxidase family are involved [67]. Although CW peroxidases belong to large multigenic families (139 members in *Medicago truncatula* [37]), only a few members have been functionally characterized in planta [67], a task made more difficult by their functional redundancy. Targeted gene expression analysis under different growth conditions provides an alternative strategy to study their role in planta. In this respect, in a recent study we identified some alfalfa class III peroxidases whose expression is affected by abiotic stresses [68].

The three MsPrx15 isoforms we identified share the highest similarity with the *A. thaliana* class III peroxidase AtPrx53. However, given the limited sequence identity of 58%, no specific function can be attributed. Unfortunately, data concerning MsPrx15, or its closest homologue MtPrx15, are scarce. In physiological conditions, a contig showing high sequence similarity to MsPrx15 (contig_12706, e-147) is upregulated in elongating internodes. Rahman et al. (2015) showed that this apoplast-located peroxidase accumulates in roots of the salt-tolerant alfalfa ecotype NM-801 following salt treatment [69]. In *Lotus japonicus*, inoculation with *Mesorhizobium loti* induced the down-regulation of the gene coding for a MtPrx15-homologue in the roots of the plant. In *M. truncatula,* leaf wounding induces a reduction in the abundance of MtPrx15, which can be recovered by application (3 min after wounding) with the redox inhibitor diphenyleneiodonium, suggesting that MtPrx15 abundance varies with the presence of reactive oxygen species [70]. This peroxidase is furthermore a target of cysteine sulfenylation, indicating that this protein may be redox-regulated [71].

Recently, sequence variations and glycosylation events occurring on three different isoforms of this peroxidase were identified [25], without identifying changes that explain the pI-shifts observed on 2D-gels. This peroxidase is the major protein present in the LiCl fraction of alfalfa stems (representing half of the total volume of all spots on the gels of the LiCl fraction). In the current study, the higher accumulation of MtPrx15-isoforms in the upper stem regions, a site of broad metabolic activity directed toward cell wall development, indicates a role in the development of the cell wall*,* probably by promoting the cross-linking of lignin precursors.

A dirigent-like protein (CaCl_2_-2570) also attains its highest accumulation in the youngest stem region. The expression of the closest alfalfa homologues of the identified protein (AGED database contig_5031 and contig_29341) are likewise higher in young, elongating stems compared to post-elongation stems. A concerted action of MtPrx15 and dirigent-like protein in lignin biosynthesis or the synthesis of lignans can be hypothesized. Overexpression of a soybean homologue resulted in an increased accumulation of lignans in *Pisum sojae* and a concomitant increase pathogen resistance [72]. The shunt of monolignols to lignans via the intervention of dirigent-like proteins may therefore increase pathogen resistance, as is found in other species [73,74]. In contrast, the two class III peroxidases MtPrx29 and MtPrx41, which accumulate more in the oldest stem regions, may exert a different role in cell wall metabolism, but this latter has not been yet elucidated.

### 4.4. Enzymes Involved in Protein Degradation/Maturation Accumulate in the Oldest Stem Region

With increasing tissue maturity, a higher accumulation of a PNGase (CaCl_2_-1013) (peptide-asparagine amidase A, glycopeptidase, N-oligosaccharide glycopeptidase) is observed. In a cell wall proteome study on *Brachypodium distachyon* grains, a similar higher abundance of PNGase was observed in more mature cell walls [75]. This protein hydrolyses *N*-glycans with α-1,3-fucosylated asparagine-bound GlcNAc, as commonly found in secreted cell wall proteins [14,76]. At least two forms of PNGases can be discerned. Cytosolic PNGase (cPNGase) hydrolyses amide linkages in glycosylated Asn-residues of misfolded proteins prior to proteosomal degradation and have an optimum activity in the neutral pH region. The plant-specific acidic PNGase (aPNGase) identified in this study has an acidic optimum pH and hydrolyses glycopeptides carrying high-mannose type or complex type N-glycans in the vacuole and cell wall [77]. The aPNGase (NCBInr gi:922395483) was found in the CaCl_2_ fraction and decreased in abundance from the lignified basal region to the younger stem segments. Alignment of this aPNGase with the one from almond and tomato (referred to as PNGase-A and PNGase-Le) revealed that the proteins share three conserved regions (Supplementary Figure S2). These may be important in determining the activity of this protein. By catalyzing the hydrolysis of *N*-glycans from glycoproteins, its accumulation in the oldest stem region suggests that free *N*-glycans are preferentially generated in the apoplasm of the oldest tissues [77]. A role in the recovery of sugars from proteins in aging tissues can thus be considered.

This hypothesis would corroborate with the higher accumulation of different endo- and exoproteases in the basal region. These include aspartic proteinase Asp1 (EGTA-1119), serine carboxypeptidase S10 (EGTA-1111), eukaryotic aspartyl protease family protein (EGTA-914), and cysteine proteinase family members (EGTA-1228, EGTA-1233, and CaCl_2_-2828). However, other cysteine-proteinase family members (including vignain-like proteins) behave differently (CaCl_2_-2269, EGTA-1296, EGTA-1450, EGTA-1451, EGTA-1452), indicating the differential regulation of members of this protein family according to the developmental maturity. A functional characterization of the different proteases identified here is currently not available. By the careful examination of the mass spectra, we were able to show that the N-terminal inhibitor domain of the eukaryotic aspartyl protease gi:657371151 (EGTA-914) and the cysteine proteinase gi:357437719 (EGTA-1228, EGTA-1233) is cleaved off (Supplementary Table S1B). The protein identified in the spots EGTA-1228 and EGTA-1233 is homologous to cysteine proteinase RD21A, a protein previously identified in the apoplastic fluid of *A. thaliana* [78], in seed mucilage [79], and in the culture medium, wherein *A. thaliana* seedlings have grown [80]. This defense-related protein is predicted to undergo a three-step maturation; cleavage of the signal peptide, cleavage of N-terminal inhibitory domain (observed as the peptide with m/z 1376 Supplementary Table S1B), and finally cleavage of the C-terminal granulin domain [81]. Although we did not identify the peptide corresponding to the latter cleavage, the position on gel (Supplementary Figure S1) and the lack of peptides covering the granulin-domain while the protease domain is well covered suggest that the protein is in its fully active form. Interestingly, RD21A homologous from other species have been found in the apoplast [82], and recently the retargeting of this protein under the influence of the cyst nematode effector 4E02 was described [83].

The abundance of chloroplastic ferritin 2 and 3 was significantly higher in the basal and intermediate regions. Ferritins are ubiquitous proteins that form complexes which can gather up to 4500 atoms of iron [84] and are of major importance for iron homeostasis. In 2009, Ravet et al. showed that ferritins mainly function as defense mechanisms against free iron-induced oxidative stress and not as storage for iron needed for plant development [85]. In *Lupinus luteus*, the accumulation of ferritin mRNA and the corresponding protein was reported in senescing nodules, supporting the idea that this protein participates in iron translocation from senescing tissues to other plant regions [86]. The presence of four (NCBInr gi:357468557) and two (NCBInr gi:357492793) GTAC motifs in the 1000 bps 5’-UTR sequence upstream of the genes coding for these two2 proteins in *M. truncatula* indicates that ferritin expression may be regulated by the SPL7 transcription factor.

Remobilization of metabolites from the mature stem region was further suggested by the higher accumulation of nine beta-amylase isoforms (NCBInr gi:3913031 (homologous with Medtr4g045667.1)), involved in starch remobilization [87] and the basal regions, in comparison with both the intermediate and the apical segments. Interestingly, the screening of the 1000 bps 5’-UTR sequence upstream of the gene coding for this protein (Medtr4g045667.1) revealed the presence of four GTAC motifs, making it also potentially dependent on SPL7 regulation. These observations suggest that copper availability affects the accumulation of proteins typically present at certain stages of stem maturity. Consequently, stem development might be partially dependent on copper availability.

We finally noted the higher abundance of two LecRK-domain containing lectins (respectively NCBInr gi:2951684 (CaCl_2_-2127, CaCl_2_-2128, EGTA-1411, LiCl-765) and gi:400180 (CaCl_2_-2193, EGTA-1390, EGTA-1420) in the cell wall fraction for the basal stem. Lectin-domain proteins are known to be involved in the defense against pathogens and herbivores [88], and ATP-binding LecRK homologues are regarded as having antimicrobial activities [89].

## 5. Conclusions

This study reveals the impact of tissue maturity on the cell wall protein-enriched subproteome extracted by a sequential extraction protocol. Stem sections at three different heights along the stem, and thus different stages of cell wall development, were studied. The strongest similarities are observed between the apical and intermediate stem sections, both of which contain a high abundance of proteins that target and modulate the structure of cell wall polymers. DUF642-containing proteins, beta-galactosidase, the polygalacturonase β-subunit, stem 28 kDa glycoproteins, and endoxyloglucan transferase appear to have an important function in the youngest regions, confirming previous data [19]. These proteins are involved in modifying carbohydrate-based polymeric backbones, therefore, their higher abundance relates to the transition from young elongating regions to mature, thickening tissues.

In the most basal, oldest stem region, proteins such as PNGase and beta-amylase accumulate, suggesting that in more mature stem tissues the cell wall proteome is more oriented towards the recycling of sugars from glycoproteins and storage molecules. By studying the cytosolic proteome of the stem [25], we previously found that defence-related proteins accumulate in the basal region. Here, we report in addition the accumulation of a defensin precursor protein, various chitinases/endochitinases, and members of the class III peroxidase-family in the most mature stem segment. These observations confirm that protein-based defence is important in mature alfalfa stem cell walls. Opposed to this, the higher accumulation of dirigent-like protein in the younger tissues suggests that defence-activities are performed by the production of lignans.

## Figures and Tables

**Table 1:** Clustering of the proteins according to the procedure described in the method section: By stem region (basal part (Base); intermediate part (Int); apical part (Apex) of the stem), the average abundance measured for each spot is given as log(2) of the standardized volume and represented by a green bar. The column “Loc” and “Type” respectively indicate the localization and protein type (soluble (Sol) and membranair (Memb)), as predicted by Deeploc. Extracellular (Extrac); cytoplasmic (Cytop); plastidial (Plast); Lysosomal/vacuolar (Lyso). Detailed identification data are given in Supplementary Table S1A for the CaCl_2_ fraction, Supplementary Table S1B for the EGTA fraction, and Supplementary Table S1C for the LiCl fraction. The functional classification of the spots that contribute significantly to two PCA dimensions are given in Supplementary Table S2D.

Cluster	Spot	gi-Number	Name	Loc	Type	Base	Int.	Apex
1	CaCl2-2127	gi|2951684	lectin [M. sativa]	Extrac	Sol	0.37	−0.20	−0.39
1	CaCl2-2128	gi|2951684	lectin [M. sativa]	Extrac	Sol	0.25	−0.30	−0.27
1	CaCl2-2193	gi|400180	RecName: Full=Truncated lectin 2	Extrac	Sol	0.34	−0.14	−0.14
1	EGTA-1167	gi|388505450	Glutelin type-A [M. truncatula]	Cytop	Sol	0.25	−0.14	−0.23
1	EGTA-1390	gi|400180	RecName: Full=Truncated lectin 2	Extrac	Sol	0.40	−0.20	−0.23
1	EGTA-1411	gi|2951684	lectin [M. sativa]	Extrac	Sol	0.20	−0.29	−0.30
1	EGTA-1420	gi|400180	RecName: Full=Truncated lectin 2	Extrac	Sol	0.19	−0.19	−0.22
1	LiCl-765	gi|2951684	lectin [M. sativa]	Extrac	Sol	0.21	−0.25	−0.23
2	CaCl2-1870	gi|388505450	Glutelin type-A [M. truncatula]	Cytop	Sol	0.20	−0.20	−0.26
2	CaCl2-2363	gi|357468557	Ferritin-3 [M. truncatula]	Plast	Sol	0.24	−0.04	−0.44
2	CaCl2-2627	gi|357492793	Ferritin-2 [M. truncatula]	Plast	Sol	0.11	−0.01	−0.40
2	EGTA-2050	gi|50320305	putative defensin 2.1 precursor [M. sativa]	Extrac	Sol	0.15	−0.14	−0.19
3	CaCl2-1013	gi|922395483	PNGase A [M. truncatula]	Extrac	Sol	0.02	−0.08	−0.17
3	CaCl2-2165	gi|228204925	chitinase class III-1 [M. sativa]	Extrac	Sol	0.04	−0.12	−0.21
3	CaCl2-2426	gi|357468557	Ferritin-3 [M. truncatula]	Plast	Sol	0.09	−0.08	−0.14
3	EGTA-1228	gi|357437719	Cysteine proteinase [M. truncatula]	Lyso	Sol	0.16	−0.12	−0.14
3	EGTA-1372	gi|228204925	chitinase class III-1 [M. sativa]	Extrac	Sol	0.03	−0.14	−0.23
3	EGTA-2117	gi|3913031	RecName: Full=Beta-amylase [M. sativa]	Cytop	Sol	0.12	−0.11	−0.20
3	EGTA-570	gi|3913031	RecName: Full=Beta-amylase [M. sativa]	Cytop	Sol	0.11	−0.10	−0.22
3	EGTA-573	gi|3913031	RecName: Full=Beta-amylase [M. sativa]	Cytop	Sol	0.09	−0.06	−0.19
3	EGTA-586	gi|3913031	RecName: Full=Beta-amylase [M. sativa]	Cytop	Sol	0.12	−0.09	−0.18
3	EGTA-587	gi|3913031	RecName: Full=Beta-amylase [M. sativa]	Cytop	Sol	0.07	−0.05	−0.14
3	EGTA-593	gi|3913031	RecName: Full=Beta-amylase [M. sativa]	Cytop	Sol	0.08	−0.13	−0.22
3	EGTA-595	gi|3913031	RecName: Full=Beta-amylase [M. sativa]	Cytop	Sol	0.05	−0.11	−0.16
3	EGTA-610	gi|3913031	RecName: Full=Beta-amylase [M. sativa]	Cytop	Sol	0.12	−0.09	−0.28
3	EGTA-615	gi|3913031	RecName: Full=Beta-amylase [M. sativa]	Cytop	Sol	0.10	−0.15	−0.23
3	EGTA-640	gi|3913032	RecName: Full=Beta-amylase [M. sativa]	Cytop	Sol	0.13	−0.07	−0.14
4	CaCl2-1716	gi|388518933	Endochitinase [M. truncatula]	Extrac	Sol	0.26	0.10	−0.12
4	CaCl2-1880	gi|357448991	endo-1,3-beta-glucosidase [M. truncatula]	Extrac	Sol	0.19	0.06	−0.08
4	CaCl2-2356	gi|357468557	Ferritin-3 [M. truncatula]	Plast	Sol	0.14	0.08	−0.20
4	CaCl2-2425	gi|357468557	Ferritin-3 [M. truncatula]	Plast	Sol	0.23	0.06	−0.32
4	CaCl2-2439	gi|357468557	Ferritin-3 [M. truncatula]	Plast	Sol	0.28	0.04	−0.21
4	CaCl2-2553	gi|357468557	Ferritin-3 [M. truncatula]	Plast	Sol	0.24	−0.04	−0.18
4	CaCl2-2827	gi|22901738	high FW vegetative storage protein	Extrac	Sol	0.21	−0.03	−0.23
4	CaCl2-2828	gi|357437721	Cysteine proteinase [M. truncatula]	Lyso	Sol	0.09	0.05	−0.13
4	EGTA-1111	gi|71534882	serine carboxypeptidase S10 [M. sativa]	Extrac	Sol	0.21	0.00	−0.16
4	EGTA-1114	gi|3913031	RecName: Full=Beta-amylase [M. sativa]	Cytop	Sol	0.18	0.06	−0.08
4	EGTA-1119	gi|50317234	Aspartic proteinase Asp1 [M. truncatula]	Extrac	Sol	0.17	0.00	−0.12
4	EGTA-1143	gi|357448997	endo-1,3-beta-glucosidase [M. truncatula]	Extrac	Sol	0.15	−0.02	−0.10
4	EGTA-1166	gi|298364452	chitinase [M. sativa]	Extrac	Sol	0.11	0.03	−0.17
4	EGTA-1168	gi|657377089	peroxidase family protein [M. truncatula]	Extrac	Sol	0.16	−0.03	−0.12
4	EGTA-1233	gi|357437719	Cysteine proteinase [M. truncatula]	Extrac	Sol	0.12	0.03	−0.14
4	EGTA-1461	gi|169147017	putative thaumatin-like protein	Extrac	Sol	0.19	0.05	−0.04
4	EGTA-1922	gi|71534922	thioredoxin h [M. sativa]	Cytop	Sol	0.12	−0.01	−0.15
4	EGTA-914	gi|657371151	eukaryotic aspartyl protease	Extrac	Sol	0.19	−0.02	−0.17
4	LiCl-1068	gi|23049900	put early nodulin-like 2 [T pratense]	CM	Memb	0.10	0.09	−0.08
5	CaCl2-1769	gi|357491415	Peroxidase [M. truncatula]	Extrac	Sol	0.40	0.29	0.11
5	CaCl2-2269	gi|3688528	pre-pro-TPE4A protein [Pisum sativum]	Lyso	Sol	−0.01	0.17	0.05
5	EGTA-1052	gi|357517805	Endochitinase [M. truncatula]	Lyso	Sol	0.18	0.07	0.01
5	EGTA-1057	gi|388494834	Endochitinase [M. truncatula]	Lyso	Sol	0.23	0.09	−0.01
5	EGTA-1450	gi|3688528	cysteine proteinase [Vicia sativa]	Lyso	Sol	0.00	0.21	0.06
5	EGTA-1451	gi|571536058	PREDICTED: vignain-like [Glycine max]	Lyso	Sol	−0.02	0.15	0.00
5	EGTA-1452	gi|30141021	cysteine protease-2 [Helianthus annuus]	Lyso	Sol	−0.04	0.14	−0.01
5	EGTA-2122	gi|357448991	endo-1,3-beta-glucosidase [M. truncatula]	Extrac	Sol	0.26	0.10	−0.03
6	LiCl-885	gi|357513969	Germin-like protein [M. truncatula]	Extrac	Sol	−0.40	−0.28	−0.08
6	LiCl-935	gi|388502800	Germin-like protein [Pisum sativum]	Extrac	Sol	−0.31	−0.25	−0.14
7	CaCl2-1593	gi|593701343	Cyclophilin 38 isoform 1 [T. cacao]	Plast	Memb	−0.15	−0.06	0.13
7	EGTA-1388	gi|357480321	Lactoylglutathione lyase [M. truncatula]	Cytop	Sol	−0.15	−0.02	0.04
7	EGTA-1480	gi|357512271	Ribose-5-phosphate isomer. [M. truncatula]	Plast	Sol	−0.18	0.01	0.18
7	EGTA-1586	gi|388502800	Germin-like protein [Pisum sativum]	Extrac	Sol	−0.23	−0.07	−0.01
7	EGTA-196	gi|357437609	Beta-galactosidase [M. truncatula]	Extrac	Sol	−0.18	0.04	0.01
7	EGTA-197	gi|357437609	Beta-galactosidase [M. truncatula]	Extrac	Sol	−0.19	0.03	0.03
7	EGTA-201	gi|357437609	Beta-galactosidase [M. truncatula]	Extrac	Sol	−0.24	0.02	0.03
7	EGTA-203	gi|357437609	Beta-galactosidase [M. truncatula]	Extrac	Sol	−0.23	0.05	0.05
7	EGTA-205	gi|357437609	Beta-galactosidase [M. truncatula]	Extrac	Sol	−0.19	0.01	0.02
7	EGTA-206	gi|357437609	Beta-galactosidase [M. truncatula]	Extrac	Sol	−0.19	0.02	0.03
7	EGTA-2120	gi|357437609	Beta-galactosidase [M. truncatula]	Extrac	Sol	−0.16	0.05	0.03
7	EGTA-214	gi|357437609	Beta-galactosidase [M. truncatula]	Extrac	Sol	−0.24	0.04	0.03
7	EGTA-215	gi|357437609	Beta-galactosidase [M. truncatula]	Extrac	Sol	−0.23	0.05	0.05
7	EGTA-216	gi|357437609	Beta-galactosidase [M. truncatula]	Extrac	Sol	−0.18	0.02	0.01
7	EGTA-220	gi|357437609	Beta-galactosidase [M. truncatula]	Extrac	Sol	−0.21	0.06	0.06
7	EGTA-221	gi|357437609	Beta-galactosidase [M. truncatula]	Extrac	Sol	−0.18	0.06	0.06
7	EGTA-222	gi|657400954	beta-like galactosidase [M. truncatula]	Extrac	Sol	−0.23	0.00	0.04
7	EGTA-240	gi|357437609	Beta-galactosidase [M. truncatula]	Extrac	Sol	−0.23	0.02	0.01
7	EGTA-272	gi|87241408	Protease-associated PA [M. truncatula]	Extrac	Sol	−0.08	−0.01	0.14
7	EGTA-326	gi|357437609	Beta-galactosidase [M. truncatula]	Extrac	Sol	-0.22	0.04	0.02
7	EGTA-333	gi|357437609	Beta-galactosidase [M. truncatula]	Extrac	Sol	−0.16	0.02	0.06
7	EGTA-341	gi|357437609	Beta-galactosidase [M. truncatula]	Extrac	Sol	−0.16	0.03	0.04
7	EGTA-342	gi|357437609	Beta-galactosidase [M. truncatula]	Extrac	Sol	−0.19	0.05	0.01
7	EGTA-379	gi|357437609	Beta-galactosidase [M. truncatula]	Extrac	Sol	−0.20	−0.02	−0.05
8	CaCl2-2612	gi|357476945	Acid beta-fructofuranosidase	Lyso	Sol	−0.16	0.02	0.27
8	CaCl2-2968	gi|388514479	Oxygen-evolving enhancer protein 2	Plast	Sol	−0.16	-0.03	0.28
8	EGTA-1296	gi|357438145	Cysteine proteinase [M. truncatula]	Lyso	Sol	−0.04	0.03	0.21
8	EGTA-1485	gi|357512271	Ribose-5-phosphate isomer. [M. truncatula]	Plast	Sol	−0.16	0.04	0.24
8	EGTA-1490	gi|357512271	Ribose-5-phosphate isomer. [M. truncatula]	Plast	Sol	-0.18	0.02	0.19
8	EGTA-1794	gi|157973737	PSI reaction center IV A [Cicer arietinum]	Plast	Sol	−0.01	0.10	0.30
8	LiCl-1156	gi|537317	peroxidase [M. sativa]	Extrac	Sol	−0.07	0.05	0.24
8	LiCl-1163	gi|537317	peroxidase [M. sativa]	Extrac	Sol	0.03	0.13	0.22
8	LiCl-300	gi|657374433	polygalacturonase non-catalytic	Extrac	Sol	0.00	0.06	0.20
8	LiCl-340	gi|537317	peroxidase [M. sativa]	Extrac	Sol	−0.06	−0.01	0.20
9	CaCl2-1292	gi|388508134	Nod factor-bind. lectin [M. truncatula]	Golgi	Memb	−0.22	−0.15	0.14
9	CaCl2-1940	gi|388502358	Xylogluc. Endotransglucosylase	Extrac	Sol	−0.31	−0.01	0.11
9	CaCl2-1945	gi|357508519	Xylogluc. Endotransglucosylase	Extrac	Sol	−0.10	0.01	0.21
9	CaCl2-2042	gi|5777611	cysteine protease [M. sativa]	Extrac	Sol	−0.02	−0.08	0.18
9	CaCl2-2511	gi|388502800	Germin-like protein [Pisum sativum]	Extrac	Sol	−0.22	−0.02	0.03
9	EGTA-1376	gi|357513539	Stem 28 kDa glycoprotein [M. truncatula]	Extrac	Sol	−0.09	−0.07	0.18
9	EGTA-1391	gi|357513539	Stem 28 kDa glycoprotein [M. truncatula]	Extrac	Sol	−0.17	−0.02	0.30
9	EGTA-1661	gi|357476945	Acid beta-fructofuranosidase	Lyso	Sol	−0.15	0.01	0.18
9	EGTA-956	gi|537313	plant/F18G18-200 protein [M. truncatula]	Extrac	Sol	−0.31	−0.09	0.24
9	EGTA-965	gi|537313	plant/F18G18-200 protein [M. truncatula]	Extrac	Sol	−0.23	−0.05	0.24
10	CaCl2-1586	gi|537313	plant/F18G18-200 protein [M. truncatula]	Extrac	Sol	−0.38	−0.14	0.37
10	CaCl2-1609	gi|537313	plant/F18G18-200 protein [M. truncatula]	Extrac	Sol	−0.45	−0.20	0.42
10	CaCl2-2570	gi|388509752	unknown [Lotus japonicus] (pfam03018)	Extrac	Sol	−0.16	−0.13	0.26

## Supplementary Materials

The following are available online at https://www.mdpi.com/2079-7737/8/3/60/s1. Table S1A–G. Protein identifications and associated mass spectrometric data. Table S2A–D. Cell wall protein selection procedure and statistical data. Supplementary Figure S1: Representative gel image for each of the fractions (CaCl_2_, EGTA, and LiCl). Supplementary Figure S2: Sequence alignment of the *M. truncatula* NCBInr gi: 922395483, the PNGase identified in this study with its homologues NCBInr gi:56405352 from almonds, and NCBInr gi:723633692 from tomatoes. Alignment was carried out with Clustal Omega http://www.ebi.ac.uk/Tools/msa/clustalo/. Data Availability: The mass spectrometry proteomics data have been deposited to the ProteomeXchange Consortium via the PRIDE partner repository (https://www.ebi.ac.uk/pride/archive/) with the dataset identifier PXD010666.
